# Low expression of G protein-coupled oestrogen receptor 1 (GPER) is associated with adverse survival of breast cancer patients

**DOI:** 10.18632/oncotarget.25408

**Published:** 2018-05-25

**Authors:** Stewart G. Martin, Marie N. Lebot, Bhudsaban Sukkarn, Graham Ball, Andrew R. Green, Emad A. Rakha, Ian O. Ellis, Sarah J. Storr

**Affiliations:** ^1^ Translational and Radiation Biology Research Group, Division of Cancer and Stem Cells, School of Medicine, University of Nottingham, Nottingham City Hospital, Nottingham, NG5 1PB, UK; ^2^ John van Geest Cancer Research Centre, School of Science and Technology, Nottingham Trent University, Clifton Campus, Nottingham, NG1 4BU, UK; ^3^ Academic Pathology, Division of Cancer and Stem Cells, School of Medicine, University of Nottingham, Nottingham City Hospital, Nottingham, NG5 1PB, UK; ^4^ Nottingham Breast Cancer Research Centre, University of Nottingham, Nottingham City Hospital, Nottingham, NG5 1PB, UK

**Keywords:** GPER, breast cancer, ER, oestrogen, prognosis

## Abstract

G protein-coupled oestrogen receptor 1 (GPER), also called G protein-coupled receptor 30 (GPR30), is attracting considerable attention for its potential role in breast cancer development and progression. Activation by oestrogen (17β-oestradiol; E2) initiates short term, non-genomic, signalling events both *in vitro* and *in vivo*. Published literature on the prognostic value of GPER protein expression in breast cancer indicates that further assessment is warranted. We show, using immunohistochemistry on a large cohort of primary invasive breast cancer patients (n=1245), that low protein expression of GPER is not only significantly associated with clinicopathological and molecular features of aggressive behaviour but also significantly associated with adverse survival of breast cancer patients. Furthermore, assessment of *GPER* mRNA levels in the METABRIC cohort (n=1980) demonstrates that low *GPER* mRNA expression is significantly associated with adverse survival of breast cancer patients. Using artificial neural networks, genes associated with *GPER* mRNA expression were identified; these included notch-4 and jagged-1. These results support the prognostic value for determination of GPER expression in breast cancer.

## INTRODUCTION

Breast cancer is the most common cancer in women, with over 1.7 million cases diagnosed worldwide in 2012 [1]. The female sex hormone, oestrogen (17β-oestradiol; E2), has an important role in breast cancer development and progression, with effects mediated through nuclear oestrogen receptors (ERα and ERβ) which act directly as transcription factors to regulate the expression of genes able to alter cell survival and growth.

G protein-coupled oestrogen receptor 1 (GPER) or G protein-coupled receptor 30 (GPR30) is a G protein-coupled receptor first cloned in 1996 [2] and first described in breast cancer in the ER positive MCF-7 cell line [3]. GPER has a potential role in breast cancer although controversies exist over its subcellular localisation, and mechanism of receptor activation [4–6]. GPER has been shown to bind E2 to initiate short term, non-genomic, signalling events both *in vitro* [7–9] and *in vivo* [10]. Expression of GPER has also been shown to be associated with ER expression and status in a number of studies [11] and to attenuate the growth of ER positive breast cancer [11]. Tamoxifen has been shown to act as a GPER agonist, and GPER has been implicated in tamoxifen resistance via its upregulation in a tamoxifen resistant breast cancer cell line which results in the activation of epidermal growth factor receptor (EGFR) [12].

GPER activation upregulates interleukin-1 receptor-1 (IL1R1) expression on breast cancer cells and interleukin (IL)-1β expression on cancer associated fibroblasts in a signalling loop to encourage invasive features of breast cancer [13]. GPER also supresses migration and angiogenesis of ER negative triple negative breast cancer by inhibiting nuclear factor (NF)-κB/interleukin (IL)-6 signals [14].

GPER expression in breast cancer has been assessed in a number of studies; however, these have proved ambiguous. High GPER protein expression is associated with increased distant disease free survival in ER-positive lymph node negative disease [15], presence of metastasis [16] and adverse relapse free survival of patients treated with tamoxifen [17]. *GPER* mRNA expression is significantly lower in tumour tissue in comparison to normal tissue, indicating that GPER acts as a tumour suppressor [18, 19]. Recently, a large assessment of *GPER* mRNA expression in 781 primary breast tumours demonstrated that high GPER expression is associated with favourable overall survival and that GPER silencing may be due to hyper-methylation of the flanking regions of the upstream CpG island [19]. However a smaller study of 167 breast cancer patients showed no association between mRNA expression and patient survival [20].

This study sought to investigate the expression levels of GPER mRNA and protein in large well characterised cohorts of breast cancer patients and assess for association with survival.

## RESULTS

### GPER protein staining location and frequency

**Figure 1:**
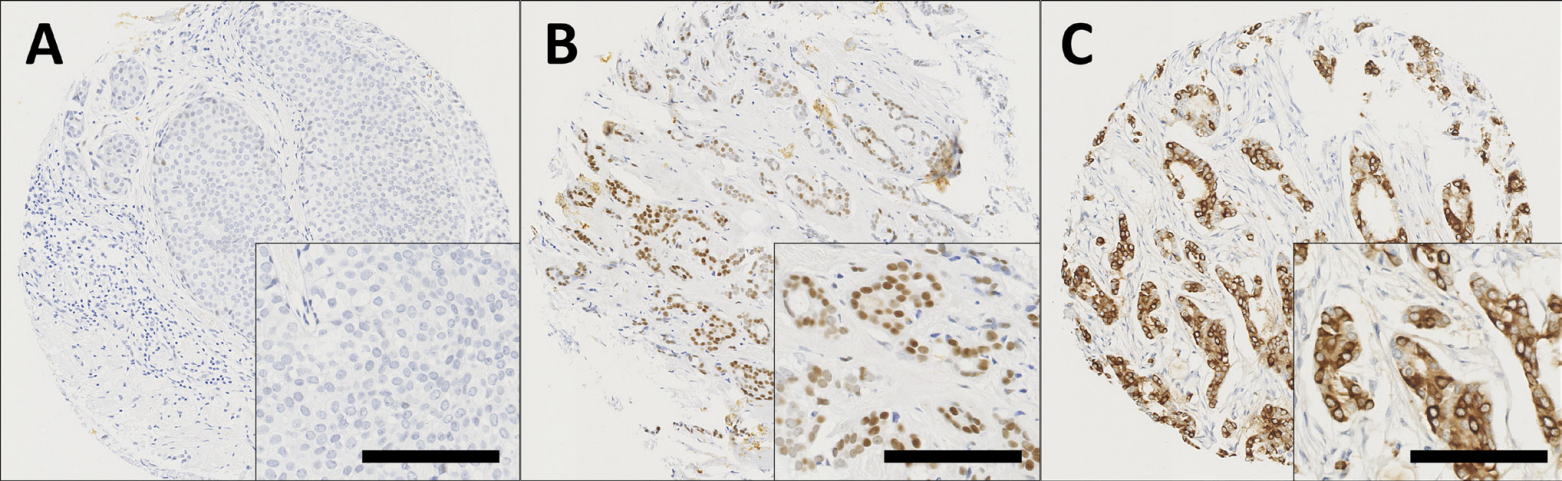
Representative photomicrographs following immunohistochemical staining of **(A)** negative nuclear and cytoplasmic GPER staining; **(B)** positive nuclear staining; **(C)** positive cytoplasmic staining in breast cancer specimens. Photomicrographs are shown at 100x magnification with 200x magnification inset box where the scale bar represents 100μm.

GPER expression was observed in both the nucleus and the cytoplasm of tumour cells. Staining varied from weak to intense, with heterogeneity observed between adjacent tumour cells. Representative photomicrographs are shown in Figure 1. Cytoplasmic GPER expression had a median H-score of 10 and ranged from 0-290. Nuclear GPER expression had a median score of 0 and ranged from 0-100. X-tile was used to generate cut points for assessment based on breast cancer specific survival with a cut point of 25 for cytoplasmic GPER expression with 73.6% of cases (916/1244) demonstrating low expression; nuclear GPER expression had a cut point of 5 with 70.0% of cases (869/1241) demonstrating low expression. A proportion of cores within the tissue microarray could not be assessed as they were missing or cores had insufficient tumour cells.

### Relationship between GPER protein expression and clinicopathological variables

High nuclear GPER expression was significantly associated with smaller tumours (χ^2^=22.5; d.f.=1; *P*<0.001), lower tumour grade (χ^2^=23.6; d.f.=2; *P*<0.001), lower NPI value (χ^2^=22.0; d.f.=2; *P*<0.001), ER positive tumours (χ^2^=4.8; d.f.=1; *P*=0.029); and with tumour stage (χ^2^=7.5; d.f.=2; *P*=0.024) (Table 1). No significant associations between cytoplasmic GPER expression and clinicopathological variables were observed (Table 1).

**Table 1:** Associations between the expression of cytoplasmic and nuclear GPER determined by immunohistochemistry with clinicopathological variables

		Cytoplasmic GPER			Nuclear GPER		
		low	high	P value	low	high	P value
Patient age	40 years or less	86 (6.9%)	30 (2.4%)	0.893	87 (7.0%)	29 (2.3%)	0.224
	Above 40 years	829 (66.7%)	298 (24.0%)		782 (63.1%)	342 (27.6%)	
Tumour size	2cm or less	538 (43.5%)	203 (16.4%)	0.349	480 (38.9)	259 (21.0%)	<0.001
	Greater than 2cm	372 (30.1%)	124 (10.0%)		384 (31.1%)	111 (9.0%)	
Tumour stage	1	559 (45.2%)	195 (15.8%)	0.608	516 (41.8%)	235 (19.0%)	0.024
	2	267 (21.6%)	105 (8.5%)		279 (22.6%)	94 (7.6%)	
	3	84 (6.8%)	27 (2.2%)		69 (5.6%)	41 (3.3%)	
Tumour grade	1	135 (10.9%)	64 (5.2%)	0.131	116 (9.4%)	83 (6.7%)	<0.001
	2	308 (24.9%)	107 (8.6%)		278 (22.5%)	135 (10.9%)	
	3	467 (37.8%)	156 (12.6%)		470 (38.1%)	152 (12.3%)	
NPI	less than 3.4	262 (21.2%)	106 (8.6%)	0.336	223 (18.1%)	144 (11.7%)	<0.001
	3.4-5.4	469 (14.3%)	636 (51.5%)		465 (37.7%)	169 (13.7%)	
	Greater than 5.4	177 (14.3%)	231 (18.7%)		175 (14.2%)	56 (4.5%)	
Basal status	Non basal	668 (57.6%)	244 (21.1%)	0.807	646 (55.8%)	264 (22.8%)	0.868
	Basal	179 (15.4%)	68 (5.9%)		174 (15.0%)	73 (6.3%)	
ER status	Negative	233 (19.4%)	94 (7.8%)	0.229	244 (20.3%)	82 (6.8%)	0.029
	Positive	655 (54.4%)	222 (18.4%)		598 (49.8%)	277 (23.1%)	
PgR status	Negative	373 (31.8%)	135 (11.5%)	0.979	369 (31.5%)	139 (11.9%)	0.218
	Positive	488 (41.6%)	176 (15.0%)		459 (39.2%)	203 (17.4%)	
HER2 status	Negative	771 (63.6%)	276 (22.8%)	0.805	725 (60.0%)	320 (26.5%)	0.105
	Positive	120 (9.9%)	45 (3.7%)		124 (10.3%)	40 (29.8%)	

The *P* values are resultant from Pearson χ^2^ test of association. ER is oestrogen receptor and PgR is progesterone receptor.

### Association between GPER protein expression and survival

**Figure 2:**
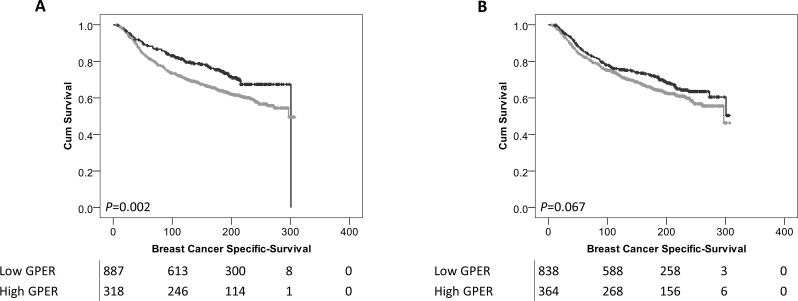
Kaplan-Meier analysis of breast cancer specific survival showing the impact of low (grey line) and high (black line) GPER protein expression within the cytoplasm **(A)** or the nucleus **(B)** with significance determined using the log-rank test. The numbers shown below the Kaplan-Meier survival curves are the number of patients at risk at the specified month.

**Figure 3:**
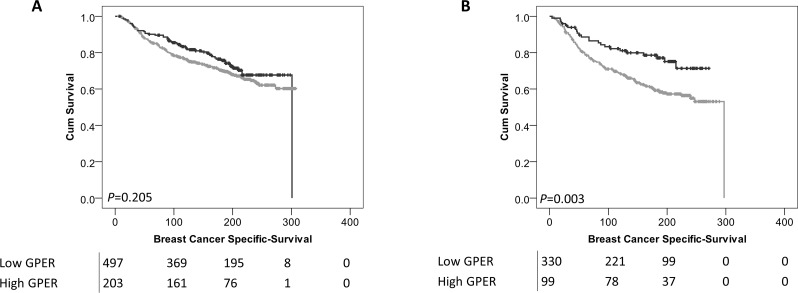
Kaplan-Meier analysis of relapse free survival showing the impact of low (grey line) and high (black line) GPER protein expression within the cytoplasm **(A)** or the nucleus **(B)** with significance determined using the log-rank test.

Low expression of cytoplasmic GPER was significantly associated with adverse breast cancer-specific survival (*P*=0.002) (Figure 2A). In multivariate Cox regression cytoplasmic GPER expression remained significantly associated (*P*=0.023) with breast cancer survival when including the potential confounding factors of tumour size, tumour stage and grade, NPI value, ER, PgR and HER2 status and Lymph node status (with individual Kaplan–Meier statistics of *P*<0.001, *P*<0.001, *P*<0.001, *P*<0.001, *P*=0.002, *P*<0.001, *P*<0.001, *P*<0.001 respectively) (Table 2). Expression of GPER in the nucleus was not significantly associated with breast cancer specific-survival (*P*=0.067) (Figure 2B). In addition to disease specific survival, cytoplasmic expression of GPER was significantly associated with adverse relapse free interval (*P*=0.023), but not nuclear GPER expression (*P*=0.057) (Figure 3A and 3B).

**Table 2:** Cox proportional hazards analysis for overall survival for cytoplasmic GPER expression in breast cancer

	*P* value	Exp(B)	95.0% CI for Exp(B)	
			Lower	Upper
Tumour size	0.031	1.373	1.030	1.831
Tumour stage	0.000	2.441	1.748	3.409
Tumour grade	0.000	1.882	1.390	2.548
NPI	0.984	1.004	0.658	1.533
ER status	0.023	1.475	1.054	2.065
PgR status	0.021	0.699	0.516	0.947
HER2 status	0.000	1.845	1.392	2.444
Lymph node status	0.081	0.701	0.471	1.045
Cytoplasmic GPER1	0.023	0.731	0.558	0.958

Exp(B) is used to denote hazard ratio and 95% CI is used to denote 95% confidence interval.

**Figure 4:**
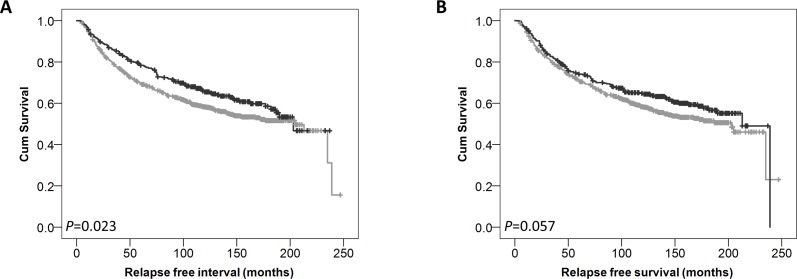
Kaplan-Meier analysis of breast cancer specific survival showing the impact of low (grey line) and high (black line) GPER protein expression within the cytoplasm in patients who did not receive endocrine therapy **(A)** or patients that did receive endocrine therapy **(B)** with significance determined using the log-rank test. The numbers shown below the Kaplan-Meier survival curves are the number of patients at risk at the specified month.

Interestingly, low expression of cytoplasmic GPER was significantly associated with adverse survival of patients who received endocrine therapy (*P*=0.003) (Figure 4B); whereas no association was observed in breast cancer-specific survival in patients who did not receive endocrine therapy (*P*=0.205) (Figure 4A). There was no difference observed in breast cancer specific survival of patients receiving endocrine therapy dependent upon nuclear GPER expression.

### *GPER* mRNA expression and clinicopathological variables

Data was available for two *GPER* probes in the Molecular Taxonomy of Breast Cancer International Consortium (METABRIC) data set: probe 1 (ILMN_1795298) and probe 2 (ILMN_2384056). Both were assessed independently for associations with clinicopathological variables and patient survival (Table 3) and were categorised into low and high groups using X-tile. Both *GPER* probe 1 and probe 2 demonstrated that low *GPER* expression were associated with basal and HER2 PAM50 subtype (χ^2^=207.4; d.f.=4; *P*<0.001 and χ^2^=177.5; d.f.=4; *P*<0.001 respectively). Low *GPER* mRNA expression was associated with P53 mutation status (χ^2^=19.7; d.f.=1; *P*<0.001 and χ^2^=33.4; d.f.=1; *P*<0.001 for probe 1 and 2 respectively), stage (χ^2^=11.4; d.f.=4; *P*=0.023 and χ^2^=11.1; d.f.=4; *P*=0.025 for probe 1 and 2 respectively), larger tumour size (χ^2^=16.2; d.f.=1; *P*<0.001 and χ^2^=16.9; d.f.=1; *P*<0.001 for probe 1 and 2 respectively), higher tumour grade (χ^2^=83.0; d.f.=2; *P*<0.001 and χ^2^=87.3; d.f.=2; *P*<0.001 for probe 1 and 2 respectively) and ER negative tumours (χ^2^=119.1; d.f.=1; *P*<0.001 and χ^2^=130.3; d.f.=1; *P*<0.001 for probe 1 and 2 respectively).

**Table 3:** Associations between the GPER mRNA expressions in the METABRIC cohort with clinicopathological variables

		GPER probe 1			GPER probe 2		
		low	high	P value	low	high	P value
PAM 50 subtype	Basal	208 (10.5%)	123 (6.2%)	<0.001	124 (6.3%)	207 (10.5%)	<0.001
	HER2	183 (9.3%)	56 (2.8%)		100 (5.1%)	139 (7.0%)	
	Luminal A	241 (12.2%)	472 (23.9%)		78 (4.0%)	637 (32.3%)	
	Luminal B	220 (11.1%)	270 (13.7%)		117 (5.9%)	372 (18.9%)	
	Normal	49 (2.5%)	150 (7.6%)		15 (0.8%)	184 (9.3%)	
P53 mutation status	Mutated	65 (8.0%)	34 (4.2%)	<0.001	49 (6.0%)	50 (6.1%)	<0.001
	Wild type	301 (36.9%)	416 (51.0%)		161 (19.7%)	557 (68.2%)	
Stage	0	234 (15.3%)	256 (16.7%)	0.023	113 (7.4%)	376 (24.6%)	0.025
	1	141 (9.2%)	229 (15.0%)		64 (4.2%)	307 (20.1%)	
	2	263 (17.2%)	308 (40.1%)		143 (9.3%)	428 (28.0%)	
	3	42 (2.7%)	48 (3.1%)		19 (1.2%)	71 (4.6%)	
	4	2 (0.1%)	8 (0.5%)		0 (0.0%)	10 (0.7%)	
Tumour size	Less than 2cm	242 (12.4)	379 (19.4%)	<0.001	102 (5.2%)	521 (26.6%)	<0.001
	2cm or greater	651 (33.2%)	686 (35.0%)		329 (16.8%)	1007 (51.4%)	
Tumour grade	1	48 (2.5%)	121 (6.4%)	<0.001	16 (0.8%)	153 (8.1%)	<0.001
	2	286 (15.1%)	483 (25.6%)		108 (5.7%)	661 (35.0%)	
	3	532 (28.2%)	419 (22.2%)		294 (15.6%)	658 (34.8%)	
ER status	Negative	301 (15.6%)	139 (7.2%)	<0.001	184 (9.5%)	256 (13.2%)	<0.001
	Positive	582 (30.1%)	913 (47.2%)		242 (12.5%)	1254 (64.8%)	

The *P* values are resultant from Pearson χ^2^ test of association. ER is oestrogen receptor and PgR is progesterone receptor.

### Association between GPER mRNA expression and patient survival

**Figure 5:**
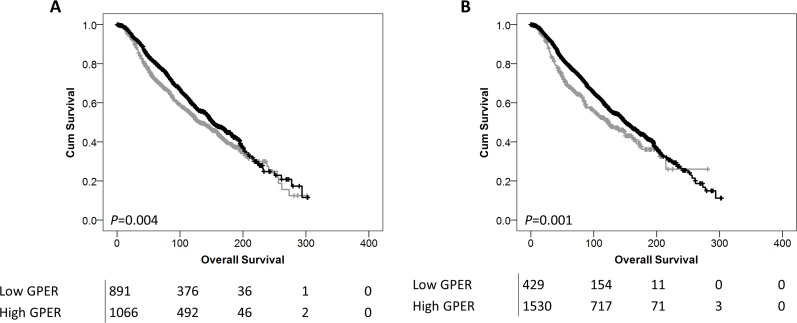
Kaplan-Meier analysis of overall survival showing the impact of low (grey line) and high (black line) *GPER* mRNA expression with probe 1 **(A)** or probe 2 **(B)** with significance determined using the log-rank test. The numbers shown below the Kaplan-Meier survival curves are the number of patients at risk at the specified month.

Low *GPER* probe 1 and probe 2 mRNA expression was significantly associated with adverse overall survival of the breast cancer cohort; (*P*=0.004) and (*P*=0.001) respectively (Figure 5A and 5B).

### Comparison between GPER mRNA and protein expression

There were 194 tumours in this study that were assessed for GPER protein expression and for *GPER* mRNA expression as part of the METABRIC cohort. Cytoplasmic expression of GPER was not correlated with *GPER* probe 1 or probe 2 mRNA expression (*P*=0.824, R^2^=-0.016 and *P*=0.868, R^2^=0.012 respectively. Nuclear GPER expression was not correlated with *GPER* probe 1 mRNA expression (*P*=0.079, R^2^=-0.126), but was correlated with GPER probe 2 mRNA expression (*P*=0.024, R^2^=0.162).

### Expression profiling

The gene expression data was analysed using an artificial neural network approach that uses a machine learning based data mining algorithm [21]. A rank order of all the genes was produced based on the minimum average root mean squared error. The top 200 transcripts were selected for *GPER* probe 1 and probe 2, and 84 common transcripts were identified. The top 20 unique transcripts include myomesin 1, ribosomal protein L39 like, vinexin beta and high density lipoprotein binding protein (Table 4). Some of these transcripts were assessed further using an ANOVA based approach to determine their relationship with *GPER* mRNA expression. A positive association was observed between both *GPER* probes and notch-4 (both *P*<0.001), jagged-1 (both *P*<0.001), claudin-5 (both *P*<0.001), CD34 (both *P*<0.001) and adenylate cyclase 4 (both *P*<0.001).

**Table 4:** The top 20 unique transcripts identified using artificial neural networks as associated with GPER mRNA expression in the METABRIC series

	Illumina ID	Gene	Description	Molecular class
1	ILMN_1680344	MYOM1	Myomesin 1	Structural protein
2	ILMN_2108357	RPL39L	Ribosomal Protein L39 Like	Ribosomal subunit
3	ILMN_1789338	SORBS3	Vinexin beta	Adhesion molecule
4	ILMN_1726210	GPIHBP1	High density lipoprotein binding protein	Unknown
5	ILMN_2172269	TMEM183B	Transmembrane protein 183B	Transcription regulatory protein
6	ILMN_1676897	HSPA12B	Heat shock 70kDa protein 12B	Heat shock protein
7	ILMN_2317581	SHANK3	SH2 and multiple ankyrin repeat domains 3	Cytoskeletal associated protein
8	ILMN_1752589	TMEM183A	Chromosome 1 open reading frame 37	Unclassified
9	ILMN_1728197	CLDN5	Claudin-5	Adhesion molecule
10	ILMN_1711157	NOTCH4	Notch-4	Cell surface receptor
11	ILMN_1672102	PTPRB	Receptor type protein tyrosine phosphatase beta	Receptor tyrosine phosphatase
12	ILMN_1748206	C20orf160	C20orf160 protein	Unclassified
13	ILMN_1738742	PLAT	Tissue type plasminogen activator	Serine protease
14	ILMN_1732799	CD34	CD34	Adhesion molecule
15	ILMN_2148944	ADCY4	Adenylate cyclase 4	Adenylate cyclase
16	ILMN_1681356	PDE2A	Phosphodiesterase 2A, cGMP-stimulated	Phosphodiesterase
17	ILMN_1719236	CDH5	Cadherin-5	Adhesion molecule
18	ILMN_1691376	JAG1	Jagged-1	Cell surface receptor
19	ILMN_1707232	EBF3	Early B-cell factor 3	Transcription factor
20	ILMN_1692340	ZNF662	FLJ45880 protein	DNA binding protein

## DISCUSSION

In this study, we describe how GPER protein and mRNA expression levels at the time of surgery are associated with breast cancer patient survival and various clinicopathological variables. Low cytoplasmic GPER protein expression was significantly associated with adverse breast cancer specific survival (*P*=0.002) and remained so in multivariate analysis including various potentially confounding factors, such as ER status. GPER expression within the nucleus was not associated with patient survival. It would be interesting to hypothesise over the importance of non-genomic actions of GPER; however, this study assessed expression of GPER with no measure of its activity.

Previously published studies have investigated GPER expression in patient samples to show a number of associations with clinicopathological variables, however the results from these have not always been in agreement. One of the largest studies to date investigated 981 primary invasive breast carcinomas, including investigation of nuclear and cytoplasmic GPER staining and is in consensus with the current findings. This study demonstrated that low expression of GPER was significantly associated with adverse patient survival and that there was no association with nuclear GPER expression and patient survival; this could not be demonstrated in multivariate analysis and was not described in any patient subset [22]. No data for systemic therapy was available for the patient cohort, so this was not assessed. Other studies have also investigated GPER expression, including a study of 481 breast cancer patients split into two cohorts of pre and post-menopausal women, which showed that high GPER protein expression was associated with increased distant disease free survival of ER positive lymph node negative and stage II breast cancer, but did not assess associations with disease specific survival [15]. A study investigating 321 invasive and 40 intraductal breast tumours showed associations between GPER expression with tumour size and the presence of distant metastasis, but also did not assess for associations with disease specific survival [16]. Furthermore GPER has been assessed in 323 breast cancer patients with a validation cohort of 103 patients to show associations between GPER expression and lymph node status, and HER2 status; this study also demonstrated an association between high GPER expression and adverse relapse free survival but no association was observed for overall survival [17].

Interestingly, we were also able to demonstrate that low cytoplasmic GPER expression was associated with adverse survival in breast cancer patients treated with endocrine therapy, mainly in the adjuvant setting. This is in disagreement with a previous study that demonstrated that high GPER expression was associated with adverse relapse free survival of breast cancer patients treated with tamoxifen but did not describe associations with breast cancer specific survival [17].

Associations between GPER protein expression and HER2 status, amongst other clinicopathological variables, have been described in some studies, however; there was no association between cytoplasmic or nuclear GPER expression with HER2 in this study. A number of associations between clinicopathological variables and nuclear GPER expression were observed, but none when expression was assessed within the cytoplasm. It is unclear as to the function of nuclear GPER expression, there are limited reports of nuclear expression *in vitro*, with studies demonstrating concentration of GPER in a compartment in close proximity to the nucleus [23], and direct nuclear localisation in breast cancer associated fibroblasts driven by changes in N-linked glycosylation [24].

We also investigated the expression of *GPER* mRNA in the METABRIC cohort. Low *GPER* expression was significantly associated with adverse survival of breast cancer patients. Two probes representing *GPER* were identified and assessed, probe 1 (ILMN_1795298) and probe 2 (ILMN_2384056), both located in the 3′ untranslated region. *GPER* mRNA expression was associated with various clinicopathological variables, the strongest association being with PAM50 subtype, ER status and tumour grade.

Other studies investigating *GPER* mRNA expression in breast cancer have done so in comparison to normal mammary tissue to demonstrate lower staining in tumour tissue [25]. One of the largest studies to date reported *GPER* expression in 84 normal breast tissues and 781 primary breast tumours using TCGA RNAseq data accessed through the UCSC Cancer Genomics Browser; they demonstrated that *GPER* expression is lower in primary tumours than normal breast tissues and that higher *GPER* expression in breast cancer patients was associated with increased survival, which is in agreement with our findings [19].

We performed artificial neural network analysis of transcriptomic array data to identify genes strongly associated with *GPER* expression. Interestingly, some well investigated proteins associated with breast cancer were identified, including notch-4, jagged-1 and CD34. Furthermore; links between some of the genes identified as associated with GPER expression have previously been described. The use of a GPER agonists has been shown to increase the levels of claudin-5 in the ischemic CA1 *in vivo* [26] and also increased levels of CD34 in mouse xenograft models of breast cancer [27]. GPER has also been shown to be capable of stimulating adenylyl cyclase activity [28]. Although a direct link with notch-4 has not been described, GPER has been shown to engage notch-1 signaling to alter gene expression and cell migration in breast cancer *in vitro* [29].

In summary, we have been able to demonstrate that low GPER protein and mRNA expression is associated with adverse survival in a large cohort of breast cancer patients. These findings suggest that GPER may have prognostic potential and may have utility as a therapeutic target and warrant further investigation in multi-centre studies.

## MATERIALS AND METHODS

### Immunohistochemistry patient cohort

This study is reported according to reporting recommendations for tumour marker prognostic studies (REMARK) criteria [30]. Ethical approval for this study was granted by Nottingham Research Ethics Committee 2, under the title ‘Development of a molecular genetic classification of breast cancer’ (C202313). 1245 early stage invasive breast cancer patients treated at Nottingham University Hospitals between 1987 and 1998 were stained for GPER protein expression. All specimens have been handled according to The Royal College of Pathologists ‘Pathology reporting of breast disease in surgical excision specimens incorporating the dataset for histological reporting of breast cancer’, with specimens sent immediately to the pathological laboratory after surgical resection and pre-dissected/incised. If incision of the fresh specimen was not possible, it was immediately placed in an adequate volume of fixative, at least twice that of the specimen.

All patients were managed in a standard manner, where all patients underwent a mastectomy or wide local excision, as decided by disease characteristics or patient choice, followed by radiotherapy if indicated. Patients received systemic adjuvant treatment on the basis of Nottingham Prognostic index (NPI), ER, and menopausal status. Patients with an NPI score less than 3.4 did not receive adjuvant treatment and patients with an NPI score of 3.4 were candidates for CMF chemotherapy (cyclophosphamide, methotrexate and 5-fluorouracil) if they were ER negative or premenopausal; and hormonal therapy if they were ER positive. Breast cancer specific survival was calculated as the time interval between primary surgery and death resultant from breast cancer.

Median survival for the cohort was 204 months as estimated by the reverse Kaplan-Meier method. The median age for this cohort was 55 years, ranging from 24 to 72. In this cohort 16.1% of patients (199/1238) had grade one tumours, 33.6% (416/1238) had grade two tumours and 50.3% (623/1238) had grade three tumours. 60.9% of patients (754/1238) had stage one tumours, 30.1% of patients (373/1238) had stage two tumours and 9.0% of patients (111/1238) had stage 3 tumours. 72.9% of patients (878/1205) were ER positive, 56.7% of patients (665/1173) were progesterone receptor (PgR) positive and 13.6% (165/1213) of patients were HER2 positive. 58.2% (725/1245) were invasive ductal carcinomas, 17.3% (215/1245) were tubular mixed, 5.9% (74/1245) were classic lobular all other subcategories accounted for less than 5% of the studied cohort.

### Immunohistochemistry

Immunohistochemistry was performed as previously described using a Novolink Polymer Detection kit (Leica) according to the manufacturers' instructions [31]. In brief, slides were deparaffinised in xylene, followed by rehydration in ethanol and water. Antigen retrieval was performed in 0.01molL-1 sodium citrate buffer (pH6.0) in a microwave for 10 minutes at 750W and 10 minutes at 450W. Tissue was treated with peroxidase block, washed with Tris-buffered saline (TBS), and then treated with protein block solution. Rabbit polyclonal anti-GPER (Thermo Scientific (PA5-28647)) was used as primary antibody diluted 1:100 and was incubated on the tissue for one hour. Tissue was washed with TBS prior to the application of post primary solution, tissue was subsequently washed with TBS and then Novolink polymer solution was applied. Immunohistochemical reactions were developed using 3, 3′ diaminobenzidine as the chromogenic substrate and tissue was counterstained with haematoxylin. Tissue was dehydrated in ethanol and fixed in xylene. Positive and negative controls were included with each staining run and were comprised of breast tumour composite sections comprising grade 1 and 2 early stage invasive tumour; negative controls had primary antibody omitted from each staining run (Supplementary Figure).

### Gene expression patient cohort

Details of the METABRIC data set (n=1980) data set have been published elsewhere [32]. For genomic and transcriptional profiling, DNA and RNA were isolated from samples and hybridised to the Affymetrix SNP 6.0 and Illumina HT-12 v3 platforms as described by Curtis et al (2012) [32]. In the METABRIC cohort ER positive and/or lymph node negative patients did not receive adjuvant chemotherapy; ER negative and/or lymph node positive patients received adjuvant chemotherapy.

### Immunohistochemistry scoring and statistical analyses

Assessment of immunohistochemical staining was conducted at 200x magnification following high resolution scanning using a Nanozoomer Digital Pathology Scanner (Hamamatsu Photonics). Staining in the cytoplasm was assessed using a semi-quantitative immunohistochemical H score; where staining intensity was assessed as none (0), weak (1), medium (2) or strong (3) over the percentage area of each staining intensity. Nuclear staining was assessed as the percentage of nuclei with any percentage intensity of staining. Greater than 30% of cores were double assessed, with both assessors blinded to clinical outcome and each other's scores. The single measure intraclass correlation coefficient were above 0.7, indicating good concordance between scorers.

Statistical analysis was performed using IBM SPSS Statistics (version 24). Data was stratified based on breast cancer specific survival using X-Tile software [33]. All differences were deemed statistically significant at the level of *P*<0.05. The Pearson χ^2^ test of association was used to determine the relationship between categorised protein expression and clinicopathological variables. Survival curves were plotted according to the Kaplan-Meier method with significance determined using the log-rank test. The primary endpoint of this study was to determine if GPER expression is associated with breast cancer specific survival.

### Identification of genes associated with GPER expression

To further understand the molecular function of GPER in human breast cancer, the METABRIC series was analysed using a supervised artificial neural network. *GPER* expression was used as the supervising variable as described by Abdel-Fatah *et al*. [21]. The artificial neural network was conducted with multi-layer perceptron architecture and sigmoidal transfer function, where weights were updated by a back propagation algorithm. The top 200 genes associated with *GPER* mRNA expression for probe 1 and probe 2 were used for further analysis.

## SUPPLEMENTARY MATERIALS FIGURE


