# Supplementary material for: Low expression of G protein-coupled oestrogen receptor 1 (GPER) is associated with adverse survival of breast cancer patients
Source: Oncotarget. 2018 May 25;9(40):25946–56. doi: 10.18632/oncotarget.25408 (PMC5995224; doi:10.18632/oncotarget.25408)
Supplement: Supplementary file 1 [file oncotarget-09-25946-s001.pdf]

Low expression of G protein-coupled oestrogen receptor 1 (GPER) is associated with adverse survival of breast cancer patients

SUPPLEMENTARY MATERIALS

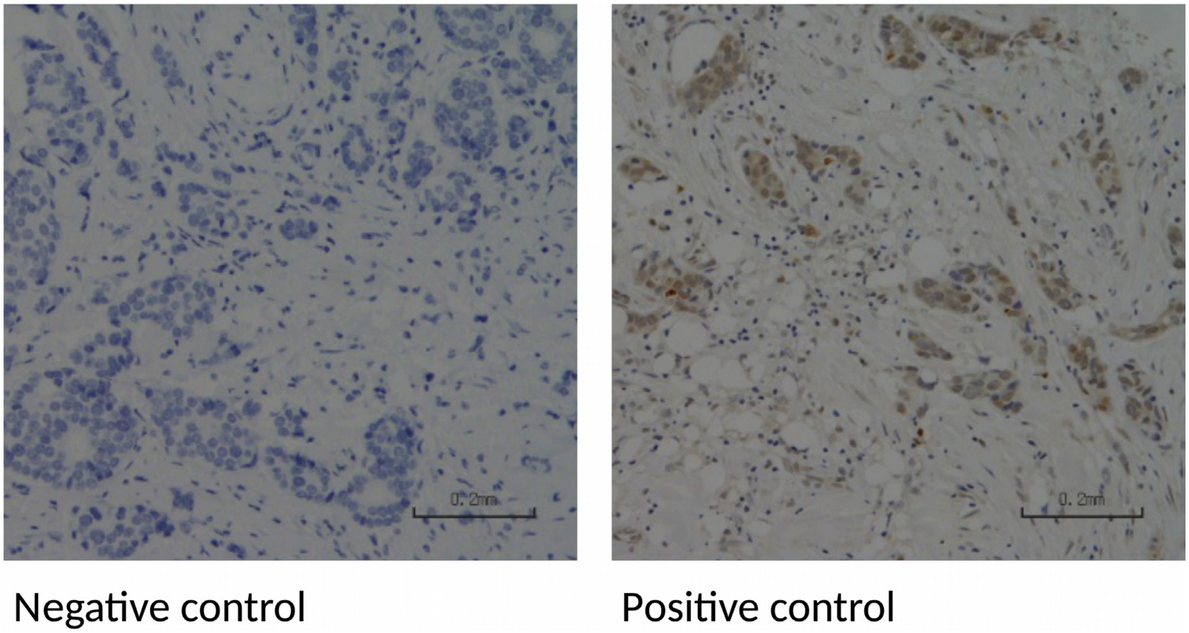

Supplementary Figure 1: Photomicrographs following immuohistochemical staining of positive and negative controls in breast cancer specimens used during GPER staining experiments.
